# Positional Signaling and Expression of *ENHANCER OF TRY AND CPC1* Are Tuned to Increase Root Hair Density in Response to Phosphate Deficiency in *Arabidopsis thaliana*

**DOI:** 10.1371/journal.pone.0075452

**Published:** 2013-10-09

**Authors:** Natasha Savage, Thomas J. W. Yang, Chung Ying Chen, Kai-Lan Lin, Nicholas A. M. Monk, Wolfgang Schmidt

**Affiliations:** 1 Institute of Integrative Biology, University of Liverpool, Liverpool, United Kingdom; 2 Institute of Plant and Microbial Biology, Academia Sinica, Taipei, Taiwan; 3 Division of Applied Mathematics, School of Mathematical Sciences, University of Nottingham, Nottingham, United Kingdom; 4 Centre for Plant Integrative Biology, School of Biosciences, University of Nottingham, Loughborough, United Kingdom; 5 Graduate Institute of Biotechnology, National Chung Hsing University, Taichung, Taiwan; 6 Genome and Systems Biology Degree Program, College of Life Science, National Taiwan University, Taipei, Taiwan; University of Michigan, United States of America

## Abstract

Phosphate (Pi) deficiency induces a multitude of responses aimed at improving the acquisition of Pi, including an increased density of root hairs. To understand the mechanisms involved in Pi deficiency-induced alterations of the root hair phenotype in Arabidopsis (*Arabidopsis thaliana*), we analyzed the patterning and length of root epidermal cells under control and Pi-deficient conditions in wild-type plants and in four mutants defective in the expression of master regulators of cell fate, *CAPRICE* (*CPC*), *ENHANCER OF TRY AND CPC 1* (*ETC1*), *WEREWOLF* (*WER*) and *SCRAMBLED* (*SCM*). From this analysis we deduced that the longitudinal cell length of root epidermal cells is dependent on the correct perception of a positional signal (‘cortical bias’) in both control and Pi-deficient plants; mutants defective in the receptor of the signal, SCM, produced short cells characteristic of root hair-forming cells (trichoblasts). Simulating the effect of cortical bias on the time-evolving probability of cell fate supports a scenario in which a compromised positional signal delays the time point at which non-hair cells opt out the default trichoblast pathway, resulting in short, trichoblast-like non-hair cells. Collectively, our data show that Pi-deficient plants increase root hair density by the formation of shorter cells, resulting in a higher frequency of hairs per unit root length, and additional trichoblast cell fate assignment via increased expression of *ETC1*.

## Introduction

Higher plants are composed of more than 40 different cell types, each equipped with a distinct set of proteins, transcripts and metabolites, enabling them to fulfill various specialized functions. All cell types were ultimately derived from totipotent stem cells in the apical meristems that are crucial for maintaining the potentially indeterminate growth of plants. In contrast to animals, almost all differentiated cells in plants posses ‘hidden’ totipotency and can, after perceiving the permissive array of signals, transdifferentiate into any cell type. This quality compensates for the sessile lifestyle of plants and allows for rapid adjustment of developmental programs after wounding, infection or in response to environmental signals.

The root epidermis of Arabidopsis has been adopted as a model to study cell fate decisions and differentiation of plant cells. Root epidermis cells derived from the meristem form a ring of 16 initials [1]. The number of cortical cells is fixed to eight, resulting in eight epidermis cells being positioned over the radial walls of cortical cells (i.e. over the cleft of two cortical cells; H position), while the other half is located over the tangential walls (N position). Cell divisions in the root epidermis occur mainly transversely, whereas longitudinal divisions only occur occasionally, serving to increase the number of epidermal cell files in later developmental stages. Root epidermal cells can enter either the hair cell fate or develop into a non-hair cell. Root hairs are long, tubular outgrowths perpendicular to the root axis, serving in the uptake of water and nutrients. In Arabidopsis, root hairs develop from epidermal cells that are located in the H position. While this pattern is not unique for Arabidopsis and has evolved at least three times independently [2], other mechanisms governing root epidermal patterning appear to be more widespread. In the majority of species, root hair patterning is random, while in others, cell fate is determined by an asymmetric division of an epidermal cell precursor [3]. Although the decision to enter the hair cell fate appears to be made at different developmental stages among these three mechanisms (positionally cued, random, and asymmetric cell division), all root hairs derive from specialized epidermal cells named trichoblasts. The term trichoblast was introduced by Leavitt [4] to describe a group of specialized cells that are typically characterized by shorter cell length, a higher rate of cell division, higher cytoplasmic density, and a smaller degree of vacuolation, characteristics that are associated with higher metabolic activity [5].

In Arabidopsis, the epidermal cell fate is conferred by an unidentified cortical signal, perceived by the LRR kinase SCRAMBLED (SCM) [6], [7]. SCM preferentially accumulates in cells in the H position and represses the expression of the *WEREWOLF* (*WER*) gene in trichoblasts. In atrichoblasts, a heterotrimeric complex consisting of a MYB domain transcription factor (either WER orMYB23), an R-like bHLH protein (either GLABRA3 (GL3) or ENHANCER OF GLABRA3 (EGL3)), and the WD40 protein TRANSPARENT TESTA GLABRA1 (TTG1), suppresses the trichoblast cell fate by promoting the expression of the homeodomain leucine zipper transcription factor *GLABRA2* (*GL2*), a negative regulator of genes initiating trichoblast differentiation [8]–[10]. Steroid hormones are required for normal expression of *WER* and *GL2* and thus for directing the cell fate of root epidermal cells [11]. The WER/MYB23-GL3/EGL3-TTG1 complex also supports the expression of the *CAPRICE* (*CPC*) and *ENHANCER OF TRY AND CPC1* (*ETC1*) genes, encoding single-repeat R3 MYB proteins, which can move between epidermal cells, probably via plasmodesmata. In trichoblasts, CPC competes with WER for binding sites, forming a new complex consisting of CPC/ETC1-GL3/EGL3-TGG1 which does not promote *GL2* expression. Lack of GL2 expression causes cells to remain in the default hair cell fate pathway [12]. Furthermore, CPC/ETC1 positively regulates SCM expression in cells in the hair position, thus reinforcing patterning [7]. The resulting spatial expression pattern of *GL2* is associated with differential conformation of chromatin at the *GL2* locus in hair files and non-hair files, which is not inherited by the daughter cells after cell divisions but re-organized upon changes in positional clues resulting from anticlinal cell divisions in the epidermis [13].

Phosphate (Pi) is an essential mineral nutrient for plants that often limits plant growth because it is relatively immobile in soils not being transported via mass flow or diffusion. As part of an amalgam of biochemical and developmental responses to Pi starvation which communally adapt the plant to low Pi availability, root hair formation is altered in a manner that increases the absorptive surface area of the root. Root hairs formed in response to Pi deficiency are substantially longer than those formed in the presence of sufficient Pi levels, contributing to the increased absorptive area of the roots under Pi-deficient conditions. Furthermore, the root epidermal cell length is reduced upon Pi deficiency, causing a higher density of root hairs [14], [15] and the formation of root hairs in positions normally occupied by non-hair cells (ectopic root hairs) has been reported in Arabidopsis [16], [17]. The molecular mechanisms which cause the alterations in root hair formation upon Pi deficiency are largely unknown. Induction of the Pi deficiency phenotype requires the concerted action of hydrolytic enzymes that mediate the rearrangement of polysaccharides and arabinogalactan proteins [18]. In addition, the ubiquitin specific protease UBP14 has been shown to be of critical importance for the Pi deficiency root hair phenotype [19]. Recently, we identified the homeodomain protein ALFIN-LIKE 6 (AL6) in a screen of T-DNA insertion lines for mutants with defects in root hair elongation under low Pi conditions as a further critical component in Pi deficiency-induced root hair formation [20]. The genes identified so far affect the length but not the density of the root hairs, suggesting that they are most likely acting downstream of the cell specification transcription factor cascade described above. How the Pi signal is integrated into intrinsic developmental programs remains enigmatic.

Using a statistical approach, mathematical modeling and well-characterized mutants defective in the expression of key cell specification genes, we show that SCM retains its influence on patterning under Pi-deficient conditions and that the characteristics that define atrichoblast cells are dependent on the perception of a positional signal via SCM. We propose that compromised perception of the positional signal acts to delay atrichoblast cell fate resolution, reducing elongation time, resulting in uniquely short, trichoblast-like cells. We further show that ETC1, in concert with shortened epidermal cells, is required for the increase in root hair density in response to Pi deficiency. We conclude that upon Pi deficiency the basic mechanisms that define cell fate remain functional; however, Pi-deficient plants increase root hair density as the result of shortening of cell length and additional trichoblast cell fate assignment.

## Results and Discussion

### The position of Pi Deficiency-induced Root Hairs is Dependent on the Correct Perception of a Positional Signal

**Figure 1:**
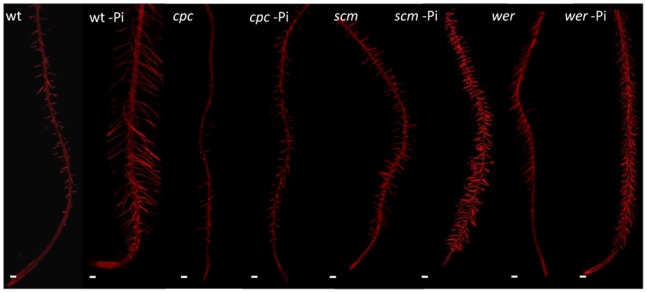
Compiled confocal micrographs of roots from wild-type (wt) plants and mutant lines with defects in the expression of genes involved in cell fate acquisition grown under control and Pi-deficient conditions. Scale bar = 150 µm.

**Figure 2:**
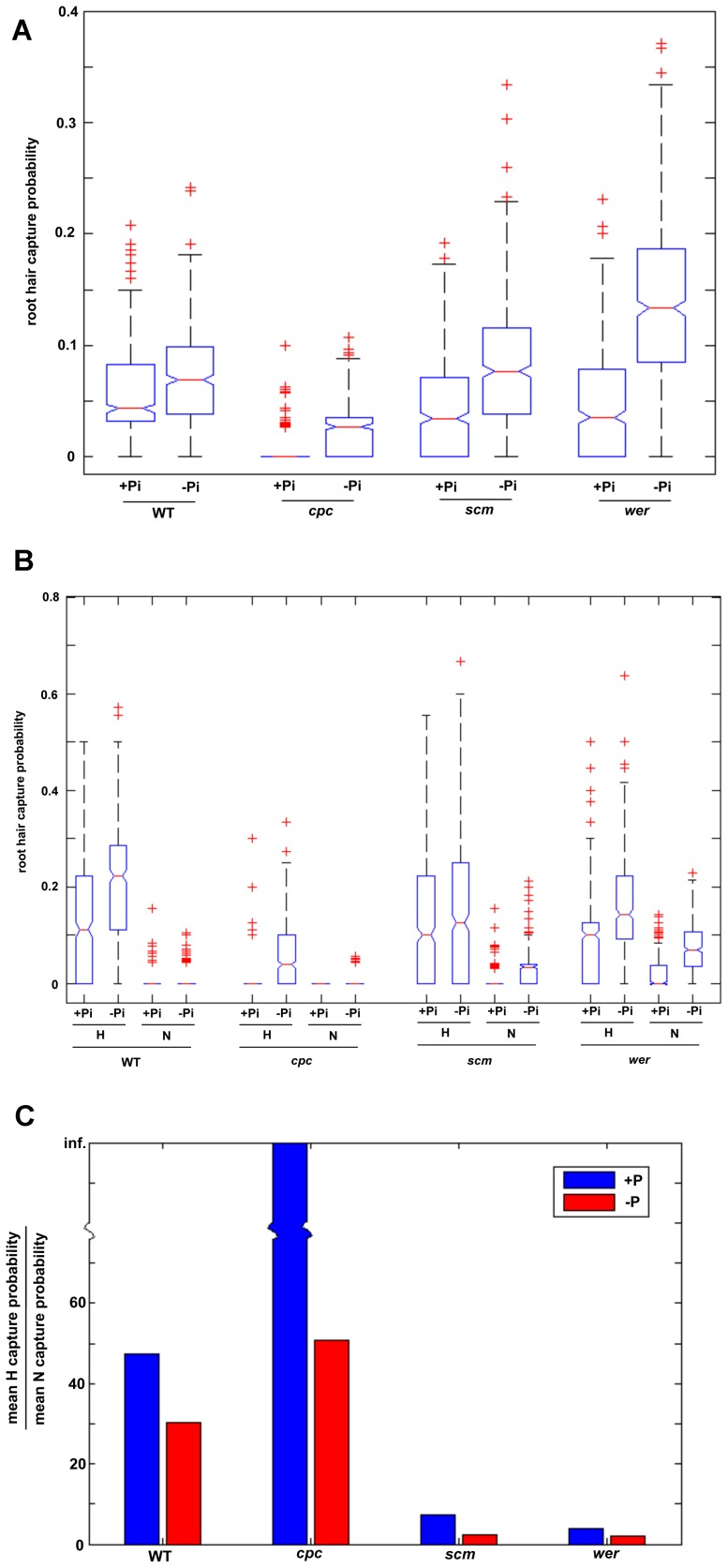
Increase in root hair density in wild-type, *cpc*, *scm* and *wer* mutant plants. A, Position-independent hair capture probability distributions. Boxplots drawn using MATLAB, 50% of the data points fall within the box, non-overlapping notches indicate the medians are significantly different with a *P* value less than or equal to 0.05. B, Position-dependent hair capture probability distributions. C, Strength of cortical bias under control (blue) and phosphate deficient (red) conditions.

An increase in the length and density of root hairs in response to Pi deprivation has been reported for a variety of plant species including Arabidopsis, and is regarded as a hallmark of Pi deficiency [16]–[21]. In Arabidopsis, root hairs are organized in longitudinal files of epidermal cells, separated by one or more files of non-hair cells [1]. Upon growth on Pi-depleted media, all backgrounds showed a marked increase in root hair length and density (Fig. 1). In principle, a higher frequency of root hairs per unit area can derive from a decreased longitudinal length of epidermal cells, an increase in the number of root hair-forming cells leading to the formation of root hairs in non-hair positions, or a combination of the two. To distinguish between these possibilities, we analyzed the number and position of root hairs and measured the longitudinal epidermal cell length of plants grown in the presence or absence of Pi. Root cross-sections, used for counting the number and position of root hairs, and cell length measurements were taken from the root hair zone, between 3 and 9 mm behind the root tip where cell elongation is completed. We calculated the probability of an epidermal cell in a cross-section having a captured hair by determining the proportion of hair-forming cells in each cross-section (Fig. 2A). This quantification of the root hair density revealed an increased probability of a cell having a captured root hair in all backgrounds upon Pi deficiency. We next asked if the increase in hair density in response to Pi deficiency was biased towards the H or N position. To answer this question, we calculated the probability of root hair formation in the H or N position by calculating the proportion of captured hair cells in the H or N position per cross-section (Fig. 2B). In backgrounds that can perceive cortical bias (wild-type and *cpc*), the increase in root hair density was almost exclusively due to an increased probability of capturing root hairs in the H position, while in backgrounds that are blind to cortical bias (*scm* and *wer*), Pi deficiency triggered root hair formation mainly in the N position. This indicates that cortical bias influences the positioning of additional root hairs formed in response to Pi deficiency.

In *scm* plants, most of the cortical bias is abolished, resulting in a compromised positioning of root hairs. Despite the loss of SCM function, root hair formation in *scm* plants is clearly biased towards the H position (Fig. 2B). This indicates that, although reduced, some cortical positional information persists, possibly due to interpretation of the signal by alternative receptors. WER is a downstream target of SCM and is thought to be the gateway for the positional signal on the patterning cascade. Thus, *wer* plants should not experience cortical bias. We found that, similar to *scm* plants, in *wer* roots the hair capture probability was greater in H positions than in N positions, suggesting that a cortical bias is also present in *wer* roots. These results suggest the existence of a SCM-independent cortical bias that influences hair cell positioning that does not act through WER, possibly acting trough MYB23.

Cortical bias was evident in all backgrounds tested under both control and Pi-deficient condition; however, more ectopic hairs were formed under the latter. We used our hair capture probability data to explore the strength of cortical bias in plants grown under control and Pi-deficient conditions. To get a measure of the strength of cortical bias, we divided the mean hair capture probability in the H position by the mean capture probability in the N position for all backgrounds. The resulting number indicated how many times more likely it is for a hair to be captured in the H position. Our data suggests that for all backgrounds cortical bias was reduced upon Pi deficiency relative to control conditions (Fig. 2C). The decrease in cortical bias might be caused by reduced signal strength, compromised detection or transduction of the signal, or by the activation of an independent mechanism counteracting the cortical signal.

### The Length of Epidermal Cells is Influenced by a Positional Signal

**Figure 3:**
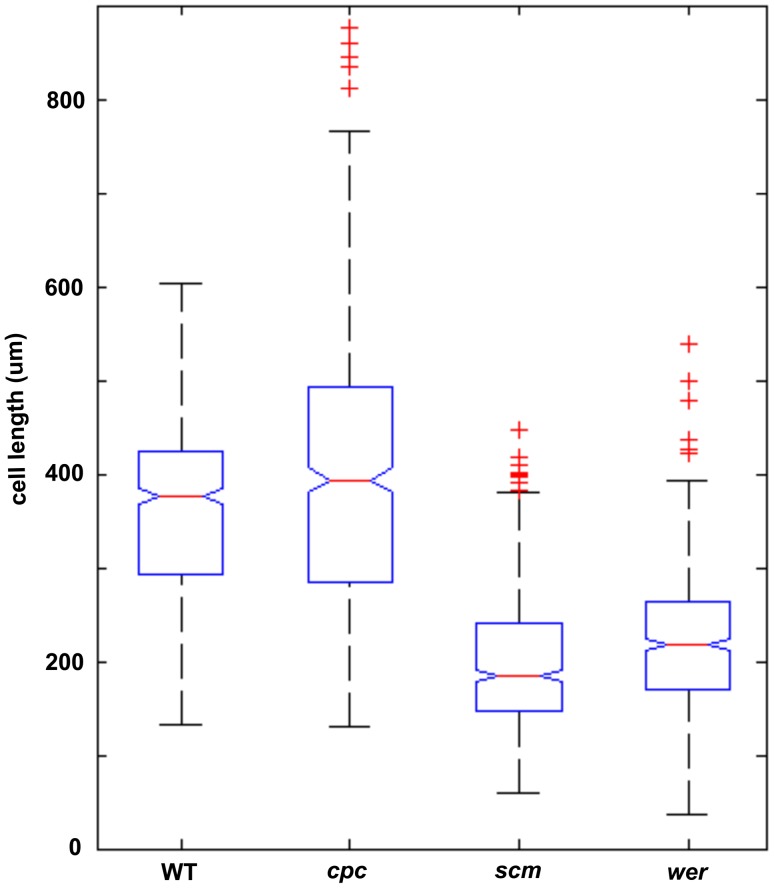
Cell length linked to cortical bias. Distributions of epidermal cell lengths for wild-type, *cpc*, *scm* and *wer* mutant plants grown under control conditions.

Current understandings states that cell length is a specific attribute characteristic to the cell type; root hair-bearing cells are short, whilst hairless cells are long [5]. As a change in longitudinal cell length would have an impact on hair density, we measured the lengths of epidermal cells from roots grown under control and Pi-deficient conditions for all backgrounds. Cell length measurements in control plants revealed that cells in the *cpc* mutant were notably longer than in *wer,* in keeping with most epidermal cells in *cpc* being atrichoblasts as opposed to trichoblast as in *wer* (Fig. 3). Based on the root hair phenotype of the *scm* mutant, a more random pattern of trichoblast and atrichoblast cell lengths would be anticipated with an average cell length that does not differ much from that of the wild-type. Unexpectedly, epidermal cells in *scm* roots were found to be shorter than cells in *wer*, resembling trichoblasts (Fig. 3). Thus, cell length does not appear to be an essential partner of cell fate. In addition, the cell length of wild-type and *cpc* (the backgrounds that can feel cortical bias) were not significantly different from each other, but they were significantly longer than both *scm* and *wer* cells (the backgrounds that cannot feel cortical bias), grouping cell length into backgrounds that are able or unable to perceive the positional signal.

### Modeling Cell Fate Suggest that Positional Information Alters the Time Evolving Probability of N-positioned Cells

**Figure 4:**
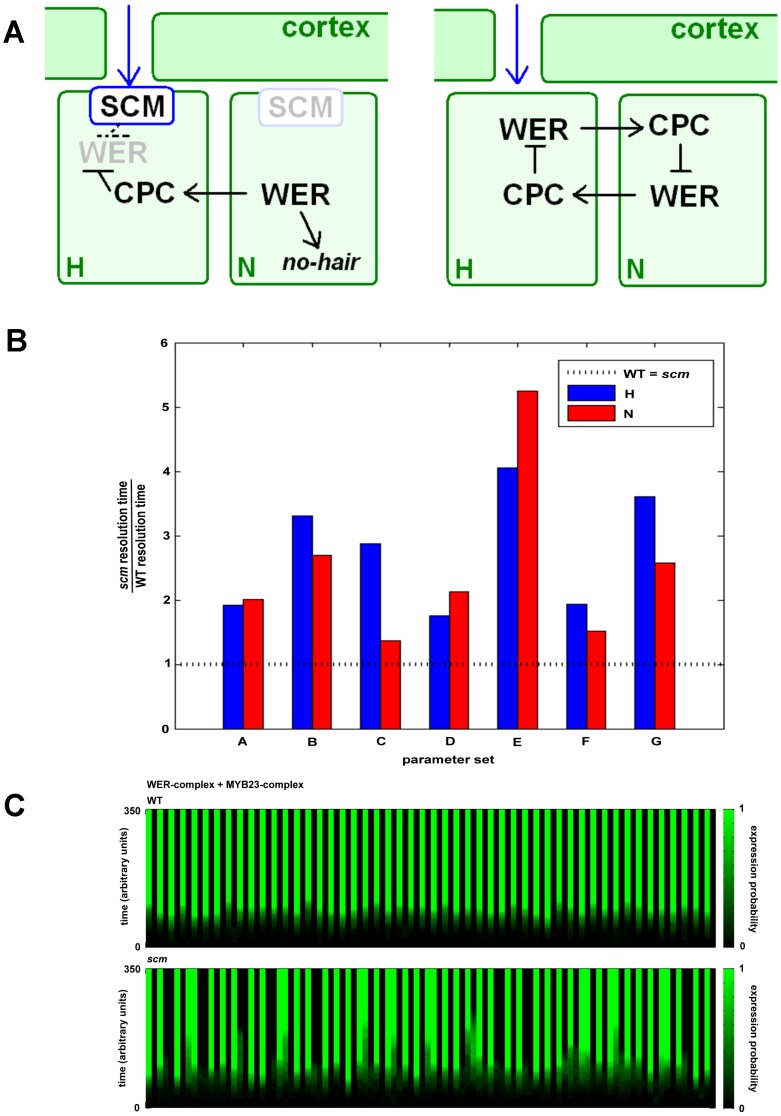
Delayed exit from default trichoblast pathway reduces atrichoblast elongation. A, Cell fate determination schematics for wild type and *scm*. B, The absence of cortical bias results in increased cell fate resolution times for simulated epidermal cells (n = 5,000) for all parameter sets tested (see Table S1). C, WER/MYB23-complex expression probability time course data for 100 cells from WT and *scm* simulations (parameter set F, Table S1). When the expression probability is 1 (green), the cell has left the default trichoblast pathway.

Experiments on cell fate acquisition suggested that the hair cell fate is the default pathway [22]. As such, the purpose of the WER patterning cascade is to facilitate the exit of epidermal cells from this default pathway. It is thought that when epidermal cells emerge from the apical meristem, all cells have an equal chance to enter the hair cell or the non-hair cell fate. During cell differentiation, two key players, WER complex and CPC complex, tussle for dominance in the individual cells. Cells in which the WER complex dominates exit the default pathway and develop into a non-hair cell. Cortical bias down-regulates *WER* transcription in the H positions, speeding competition by giving the CPC complex a head start in those cells (Fig. 4). In the absence of cortical bias, the scales of battle must be tipped by the noise intrinsic to biological processes.

An intriguing possible scenario that could account for the reduced length of N-positioned epidermal cells in *scm* plants centers on the time at which non-hair cells opt out of the default (trichoblast) pathway. We assume that under normal conditions, this decision is made before cells enter the elongation zone. If adoption of the non-hair cell fate is delayed to a time when cells have entered the elongation zone, then those cells have less time during which to elongate, and will therefore have a shorter length when exiting the elongation zone. We propose that cortical bias speeds cell fate determination by tipping the scales in favor of non-hair fate in the N positions. If this is so, then a reduction in cortical signal perception will delay the resolution of non-hair cell fate. To evaluate this scenario, we used the mathematical model for cell fate determination described by Savage et al. [23] with the addition of MYB23, which is now understood to be an integral component of the cell fate patterning network [10]. *MYB*23 is up-regulated by the WER complex. It is thought that MYB23 can bind GL3/EGL3-TTG1 to form the MYB23 complex, which has the same function as the WER complex. Thus, the MYB23 complex is part of a positive feedback loop promoting *GL2* expression and the non-hair cell fate. The model epidermis is composed of a ring of identical cells, each of which has the ability to realize all key interactions involved in cell fate acquisition as described in previous experimental studies. The existence of underlying cortical cells is implicit in the model, such that H and N positions alternate in the epidermal ring. The model employs a Boolean formalism, with two stochastic components per cell-net, *WER* mRNA and the WER/MYB23 complex, which have time-evolving probabilistic expression, providing a logic-based framework for exploring the consequences of specific assumptions on epidermal patterning with a minimal requirement for parameter specification (see [23] and Materials and Methods for details). Simulating the effect of cortical bias on the time-evolving probability of the WER/MYB23 complex, we find that non-hair cells in *scm* roots take between 1.4 and 5.3 times longer for cell fate to resolve than in wild-type plants (Fig. 4). These results support the idea that in *scm* roots non-hair cells may opt out of the default trichoblast fate after entering the elongation zone, reducing their elongation time. While this scenario implies that SCM also affects the resolution of the hair cell fate, this will not translate into an altered cell length since these cells have entered (and will not leave) the default pathway, making their resolution time irrelevant. Such a decoupling of cell identity and root hair formation resembles the effect of hormones, which act after cell patterning has been established by the WER/MYB23-GL3/EGL3-GL2 cascade in the meristem [24].

**Figure 5:**
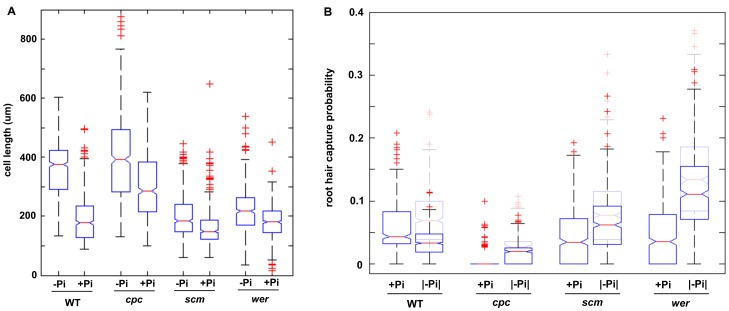
Reduction in cell length is not sufficient to account for increased hair density under Pi-deficient conditions. A, Distributions of epidermal cell lengths for wild-type, *cpc*, *scm* and *wer* mutant plants grown under control and Pi-deficient conditions. B, Root hair capture probability distributions for plants grown under control conditions (+Pi) compared with normalized capture probabilities for plants grown under Pi-deficient conditions (|−Pi|, dark blue box plots). The un-normalized capture probabilities for plants grown under Pi-deficient conditions are shown in light blue for comparison.

The above ‘delayed exit from default pathway’ hypothesis can account for the cell length grouping in the backgrounds tested under control conditions (Fig. 3). That is, in backgrounds that can perceive cortical bias, cell fate is established more rapidly and atrichoblast elongation time is maximized. This hypothesis predicts that, as Pi-deficient plants exhibit a reduction in H position bias (Fig. 2C), fate determination should be delayed under Pi-deficient conditions, giving non-hair cells a reduced elongation time leading to shorter cells. Indeed, we found that the longitudinal epidermal cell length of all genotypes was significantly reduced upon Pi deficiency (Fig. 5A). The Pi deficiency-induced decrease in longitudinal cell length was much less pronounced in *wer* and *scm* plants, possibly because most cells in this background are already close to a minimal cell length defined in the default trichoblast pathway.

### Cell Length Reduction is not Sufficient to Account for Hair Density Increase in Roots of Pi-deficient Plants

To address the question of whether cell length reduction can account for the increase in hair capture probability under Pi-deficient (−Pi) conditions, we calculated how many –Pi cell lengths there are per control (+Pi) cell length using the median cell length data shown in Figure 5A. Multiplying all –Pi capture probability distributions (Fig. 2A) by this factor gives a –Pi capture probability normalized for cell length (Fig. 5B). Comparing the normalized –Pi capture probability distributions (denoted |−Pi|, Fig. 5B) with the +Pi distributions, we see that while all –Pi capture probability distributions were reduced by the normalization, only reduction of the −Pi median in wild-type plants is sufficient to compare with the +Pi median (Fig. 5B). Given that under both control and Pi-deficient conditions, root hairs in wild-type plants tend to grow in files that are located over the cortical clefts (Fig. 2B), the results illustrated in Figure 5B suggest that in wild-type plants the increase in root hairs under Pi-deficient conditions can be accounted for chiefly, if not entirely, by a decrease in cell length along these files, rather than by the occupation of N positions. However, in all mutant backgrounds tested, the reduction in cell length under Pi-deficient conditions did not account for the increased capture of root hairs, indicating that an additional mechanism for increasing the root hair density is being employed, enabling the formation of root hairs in positions normally occupied by non-hair cells.

### ETC1 Promotes Root Hair Cell Fate Under Pi-deficient Conditions

**Figure 6:**
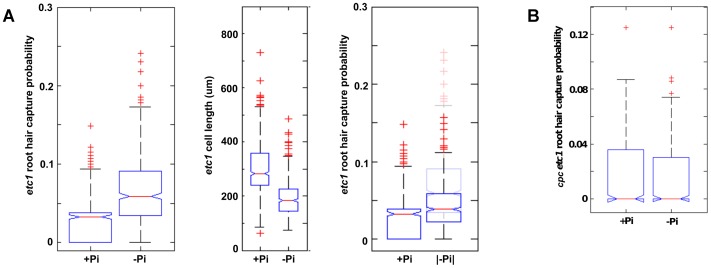
ETC1 provides an additional mechanism for increasing hair density under Pi-deficient conditions. A, (Left) Root hair capture probability for *etc1* plants grown under control and Pi-deficient conditions. A, (Middle) Cell length distributions for *etc1* plants. A, (Right) Capture probability for *etc1* plants grown under control conditions compared with the normalized capture probability of *etc1*–Pi plants. B, Root hair capture probability for *cpc etc1* plants grown under control and Pi-deficient conditions.

RNA sequencing of roots grown under Pi-deficient and control conditions revealed that *ETC1* was robustly up-regulated (3.8-fold) upon Pi deficiency, whereas the expression of all other key players in cell specification (i.e. *WER*, *SCM*, *CPC*, *GL3*, *EGL3*, *GL2*) was not significantly affected by Pi deficiency (Fig. S1; [25]). Previous studies have shown that ectopic expression of *ETC1* caused excessive root hair formation [26]. We further showed recently that *ETC1* is a putative target of the homeodomain protein ALFIN LIKE 6, which is essential for the elongation of Pi deficiency-induced root hairs [20]. It thus seems reasonable to ask if ETC1 is involved in the secondary mechanism, working alongside cell length reduction with the aim of increasing root hair density under Pi-deficient conditions. If this were true, then any increase in hair density under Pi-deficient conditions in *etc1* plants would be solely due to cell length reduction. This was indeed found to be the case (Fig. 6A). In addition, we looked at the *cpc etc1* double mutant, the prediction being that, unlike a mutation in *CPC* alone, any increase in root hair density under Pi-deficient conditions should be completely accounted for by a decrease in cell length, as the ETC1 density-increasing mechanism is no longer operative. In fact, there was no increase in hair capture probability under Pi-deficient conditions in this background (Fig. 6B), indicating that ETC1 is required for inducing the root hair phenotype typical of Pi-deficient plants.

## Methods

### Plants

Arabidopsis (*Arabidopsis thaliana* L.) plants were grown in a growth chamber on an agar-based medium as described by Estelle and Somerville [27]. Seeds of the *wer, scm-2* and *cpc etc1* double mutants were kindly provided by John Schiefelbein (University of Michigan, Ann Arbor). The other genetic stocks were obtained from the Arabidopsis Biological Resource Center (ABRC, Ohio State University, Columbus). All mutants have been described elsewhere: *cpc* [28] *wer* [8], *scm-2* [6], *etc1* [26]. Seeds were surface-sterilized by immersing them in 5% (v/v) NaOCl for 5 min and 96% ethanol for 7 min, followed by four rinses in sterile water. Seeds were placed onto Petri dishes and kept for 1 d at 4°C in the dark, before the plates were transferred to a growth chamber and grown at 21°C under continuous illumination (50 µmol m^−2 ^s^−1^, Phillips TL lamps). The medium was composed of (mM): KNO_3_ (5), MgSO_4_ (2), Ca (NO_3_)_2_ (2), KH_2_PO_4_ (2.5), (µM): H_3_BO_3_ (70), MnCl_2_ (14), ZnSO_4_ (1), CuSO_4_ (0.5), NaCl (10), Na_2_MoO_4_ (0.2) and 40 µM FeEDTA, solidified with 0.3% Phytagel (Sigma-Aldrich). Sucrose (43 mM) and 4.7 mM Mes were included and the pH was adjusted to 5.5. Ten days after sowing, plants were transferred to fresh agar media (control plants) or media without Pi. The lower concentration of K because of the absence of KH_2_PO_4_ in the Pi-free media was corrected by the addition of KCl. Plants were analyzed 6 d after replanting to the different growth conditions.

### Confocal Microscopy and Cell Length Measurements

Plants were placed in 10 mg/ml propidium iodide solution (PI) for one minute and gently rinsed with water for two minutes. The root was removed and mounted in fresh water. The roots where then observed using a confocal laser scanning microscope (Zeiss LSM510 Meta). The peak excitation λ and emission λ for PI was 536 nm and 620 nm, respectively.

The cell length of trichoblasts and atrichoblasts was measured using ImageJ (http://rsb.info.nih.gov/ij/). The position of each cell was calculated from the cumulative length of all cells between the cell and the quiescent center. The data sets were then smoothed and interpolated into 25-mm-spaced data points using a kernel-smoothing routine [29], this was performed using a Microsoft Excel macro, which enable the average calculation between replicate roots.

### Light Microscopy

The position and number of root hairs was examined in 45 to 50 30 µM cross-sections from 10 roots for each genotype and treatment (a total of 4,500 to 5,000 cross-sections for the five genotypes and two growth conditions). To avoid counting the same root hair twice, every fourth section from serial cuttings was analyzed. The root samples were fixed, dehydrated, and then embedded in Technovit 7100 (Heraeus Kulzer, Wehrheim) resin in gelatine capsules. Transverse sections (30 µm) were cut using a RM 2255 Leica microtome (Leica, Nussloch, Germany). Sections were dried and stained with toluidine blue (0.05%) on glass slides and examined using bright-field on an Imager Z1 microscope (Zeiss, Jena, Germany).

### Modeling

We modified a previously published modeling framework for Arabidopsis epidermal cell fate determination [23] to include MYB23, which is now understood to be an integral part of cell fate resolution [10]. Mechanisms encoded in the model are:

- The SCRAMBLED receptor (denoted SCM in the model) receives the cortical signal in cells in the H position [6].
- WEREWOLF mRNA (denoted *WER*) has a basal transcription rate (*c*_1_), which is down-regulated by the SCRAMBLED receptor when a cortical signal is present (*c*_0_) [30] and by the protein CAPRICE (denoted CPCp, *c*_2_; [31]).
- The WEREWOLF/MYB23 complex (WERc) is formed by WEREWOLF (WERp, *c*_3_) or MYB23 (MYB23p, *c*_6_) binding GLABRA3 (GL3p). CAPRICE protein (CPC) can also bind GLABRA3 (*c*_4_), reducing its availability to WERWOLF and MYB23 [32], [10]. The WERWOLF/MYB23 complex has basal degradation (*c*_5_).
- The WEREWOLF/MYB23 complex up-regulates the transcription of CAPRICE (*CPC*) and MYB23 (*MYB23*), and down-regulates the transcription of GLABRA3 (*GL3*) [31], [33], [34], [10]. Thus, if the WEREWOLF/MYB23 complex is present in a cell at time t, CAPRICE and MYB23 mRNA are present at time t+1, and GLABRA3 mRNA is not.
- If WEREWOLF/MYB23 mRNA is present in the cell at time t, then the WEREWOLF/MYB23 protein is present at time t+1.
- As both GLABRA3 and CAPRICE proteins are known to move out of the cells in which they were transcribed [35], [36], a cell will contain GLABRA3, CPC protein at time t+1 if its neighboring cells contain GLABRA3, CPC mRNA at time t.

The modified model is described by the equations
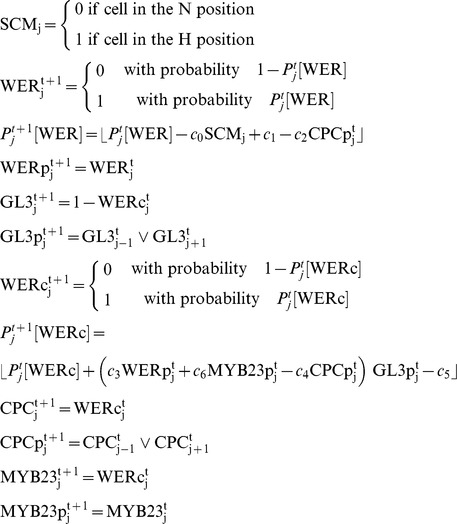



The subscript *j* and the superscript *t* represent the cell index and time, respectively. Expressions within the brackets 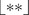 are held on the unit interval, i.e. 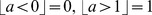. 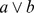 is the logical OR operation.

The accumulation of WER-complex is the key determinant of atrichoblast cell fate, and thus the exit from the default trichoblast pathway. It is thought that CPC inhibits WER complex formation by competitive binding of GL3/EGL3/TTG. The WER-complex positively regulates MYB23, and it has been shown that MYB23 has the same function as WER. We therefore assume that MYB23 competes with WER for GL3/EGL3/TTG binding. As little is known about the relative binding strengths of WER, MYB23 and CPC with regard to complex formation, we modelled a range of complex formation binding strengths (Table S1; Fig. 4B).

## Conclusions

Current understanding posits that cell length is a specific attribute characteristic to the two cell types found in the root epidermis: short cells become root hair-bearing cells, whilst long cells remain hairless. We report here that analysis of patterning mutants and plants subjected to Pi starvation challenge this strict distinction and provide evidence to suggest that cell fate determination is not necessarily linked to cell length, thereby conferring plasticity to cell fate decisions after cells exit the meristem. We further hypothesize that a lack of perception of cortical biasing signals delays cell fate determination, reducing the time during which atrichoblasts can undergo elongation, resulting in shorter non-hair cells. Our data further support a key role of ETC1 in root hair formation under Pi-deficient conditions, acting in concert with CPC1 to promote an increased frequency of root hairs.

## Supporting Information

Figure S1 **Changes in the expression of cell specification genes in response to Pi deficiency.**
(DOCX)

Table S1 **Complex binding strengths and cell fate resolution time.**
(DOCX)
