# Supplementary material for: Positional Signaling and Expression of ENHANCER OF TRY AND CPC1 Are Tuned to Increase Root Hair Density in Response to Phosphate Deficiency in Arabidopsis thaliana
Source: PLoS One. 2013 Oct 9;8(10):e75452. doi: 10.1371/journal.pone.0075452 (PMC3794009; doi:10.1371/journal.pone.0075452)
Supplement: Figure S1 — Changes in the expression of cell specification genes in response to Pi deficiency. (DOCX) [file pone.0075452.s001.docx]

**Figure S1.** Changes in the expression of cell specification genes in response to Pi deficiency. Data are taken from Lan et al. (2012).
