# Supplementary material for: Positional Signaling and Expression of ENHANCER OF TRY AND CPC1 Are Tuned to Increase Root Hair Density in Response to Phosphate Deficiency in Arabidopsis thaliana
Source: PLoS One. 2013 Oct 9;8(10):e75452. doi: 10.1371/journal.pone.0075452 (PMC3794009; doi:10.1371/journal.pone.0075452)
Supplement: Table S1 — Complex binding strengths and cell fate resolution time. (DOCX) [file pone.0075452.s002.docx]

**Table S1.** Complex binding strengths and cell fate resolution time. Grey cells show parameters used in Savage et al. (2008). Red and Blue cells correspond to bars in Figure 4B. WT, wild-type.

| $c_{0}=0.01$  $c_{1}=0.01$  $c_{2}=0.015$ | binding strength | | | time of cell fate resolution  (arbitrary units) | | | |  | |
| --- | --- | --- | --- | --- | --- | --- | --- | --- | --- |
| $c_{5}=0.01$ |  | | | WT | | *scm* | | *scm*/WT | |
| parameter  set | WER-complex  Formation  ($c_{3}$) | CPC-complex  Formation  ($c_{4}$) | MYB23-complex  Formation  ($c_{6}$) | H | N | H | N | H | N |
| A | 0.03 | 0.03 | 0.03 | 158 | 178 | 304 | 357 | 1.92 | 2.01 |
| B | 0.015 | 0.015 | 0.015 | 122 | 157 | 403 | 424 | 3.30 | 2.70 |
| C | 0.011 | 0.011 | 0.011 | 217 | 334 | 625 | 458 | 2.88 | 1.37 |
| D | 0.015 | 0.03 | 0.015 | 166 | 129 | 290 | 274 | 1.75 | 2.12 |
| E | 0.015 | 0.01 | 0.015 | 156 | 119 | 632 | 625 | 4.05 | 5.25 |
| F | 0.015 | 0.015 | 0.03 | 169 | 183 | 326 | 279 | 1.93 | 1.52 |
| G | 0.015 | 0.015 | 0.01 | 129 | 169 | 465 | 434 | 3.60 | 2.57 |
